# Association between body mass index and 1-year outcome after acute myocardial infarction

**DOI:** 10.1371/journal.pone.0217525

**Published:** 2019-06-14

**Authors:** Dae-Won Kim, Sung-Ho Her, Ha Wook Park, Mahn-Won Park, Kiyuk Chang, Wook Sung Chung, Ki Bae Seung, Tae Hoon Ahn, Myung Ho Jeong, Seung-Woon Rha, Hyo-Soo Kim, Hyeon Cheol Gwon, In Whan Seong, Kyung Kuk Hwang, Shung Chull Chae, Kwon-Bae Kim, Young Jo Kim, Kwang Soo Cha, Seok Kyu Oh, Jei Keon Chae, on behalf of KAMIR-NIH registry investigators

**Affiliations:** 1 The Catholic University of Korea, Daejeon St. Mary’s Hospital, Seoul, Republic of Korea; 2 The Catholic University of Korea, Seoul St. Mary's Hospital, Seoul, Republic of Korea; 3 Gachon University, Gil Medical Center, Incheon, Republic of Korea; 4 Chonnam National University Hospital, Gwangju, Republic of Korea; 5 Korea University, Guro Hospital, Seoul, Republic of Korea; 6 Seoul National University Hospital, Seoul, Republic of Korea; 7 Sungkyunkwan Universtiy, Samsung Medical Center, Seoul, Republic of Korea; 8 Chungnam National University Hospital, Daejeon, Republic of Korea; 9 Chungbuk National University Hospital, Cheongju, Republic of Korea; 10 Kyungpook National University Hospital, Daegu, Republic of Korea; 11 Keimyung University Dongsan Medical Center, Daegu, Republic of Korea; 12 Yeungnam University Hospital, Daegu, Republic of Korea; 13 Pusan National University Hospital, Busan, Republic of Korea; 14 Wonkwang University Hospital, Iksan, Republic of Korea; 15 Chonbuk National University Hospital, Jeonju, Republic of Korea; Azienda Ospedaliero Universitaria Careggi, ITALY

## Abstract

**Objectives:**

Beneficial effects of overweight and obesity on mortality after acute myocardial infarction (AMI) have been described as **“**Body Mass Index (BMI) paradox”. However, the effects of BMI is still on debate. We analyzed the association between BMI and 1-year clinical outcomes after AMI.

**Methods:**

Among 13,104 AMI patients registered in Korea Acute Myocardial Infarction Registry-National Institute of Health (KAMIR-NIH) between November 2011 and December 2015, 10,568 patients who eligible for this study were classified into 3 groups according to BMI (Group 1; < 22 kg/m^2^, 22 ≤ Group 2 < 26 kg/m^2^, Group 3; ≥ 26 kg/m^2^). The primary end point was all cause death at 1 year.

**Results:**

Over the median follow-up of 12 months, the event of primary end point occurred more frequently in the Group 1 patients than in the Group 3 patients (primary endpoint: adjusted hazard ratio [aHR], 1.537; 95% confidence interval [CI] 1.177 to 2.007, p = 0.002). Especially, cardiac death played a major role in this effect (aHR, 1.548; 95% confidence interval [CI] 1.128 to 2.124, p = 0.007).

**Conclusions:**

Higher BMI appeared to be good prognostic factor on 1-year all cause death after AMI. This result suggests that higher BMI or obesity might confer a protective advantage over the life-quality after AMI.

## Introduction

The prevalence and socio-economic influence of obesity are dramatically increasing over the globe. In general, obesity is well known to be related to aggravated cardiovascular disease [1–6]. However, several studies showed restrictive impacts on cardiovascular outcomes in patients undergoing percutaneous coronary intervention (PCI) [7–9]. Clinical effects of body mass index (BMI) after PCI in acute myocardial infarction (AMI) are still controversial. Some previous reports revealed that obesity paradox was observed but not reached to the significant difference after the multivariate analysis [10, 11], whereas other studies showed that obese patients with AMI had an improved prognosis after primary PCI [9, 12, 13]. Therefore, the aim of the present study ought to evaluate the clinical outcome in patients with AMI undergoing PCI in accordance with weight status.

## Methods

### Study design and population

**Fig 1:**
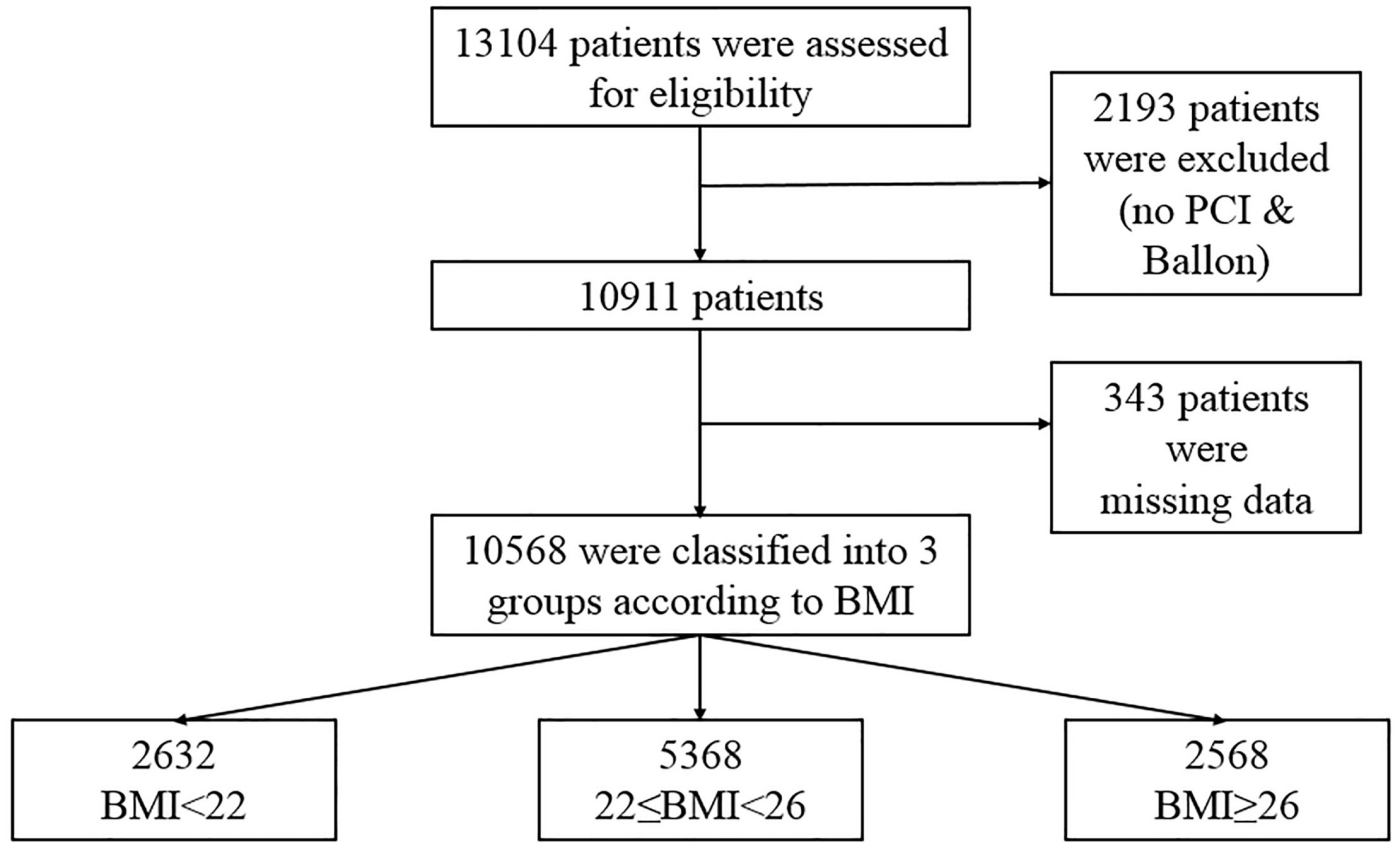
Overview of the study population. BMI = body mass index; PCI = percutaneous coronary intervention.

Fig 1 is a flow chart of this study. We consecutively enrolled 13,104 patients with AMI who had successful PCI from the database of the Korea Acute Myocardial Infarction Registry–National Institutes of Health (KAMIR-NIH). The KAMIR-NIH is a prospective, multicenter, web-based observational cohort study to develop the prognostic and surveillance index of Korean patients with AMI from 15 centers in Korea and was supported by a grant of Korea Centers for Disease Control and Prevention from May 2010 to June 2015. Among them, 2,193 patients who did not undergo PCI or PTCA and 343 patients with missing data were excluded. The remaining 10,568 patients were divided into three groups according to the BMI.

All participated centers are high-volume centers for coronary angiography with PCI and have used the same study protocol. This study was approved by the institutional review board (IRB) of each participating institution (IRB of the Catholic University of Korea, Daejeon, St. Mary’s hospital, IRB of the Catholic University of Korea, Seoul, St. Mary’s hospital, Gachon Gil Medical Center, IRB of Chonnam National University, Korea University Guro IVD Support Center, Seoul National University Hospital Biomedical Research Institute, Samsung Medical Center Clinical Trial Center, IRB of Chungnam National University Hospital, IRB of Chungbuk National University Hospital, IRB of Kyungpook National University Hospital, Clinical Trial Center of Keimyung University Dongsan Medical Center, Clinical Trial Center for Medical Devices of Yeungnam University Hospital, Clinical Trial Center of Pusan National University Hospital, IRB of Wonkwang University Hospital and IRB of Chonbuk National University Hospital) and was conducted according to the Declaration of Helsinki with patients’ written informed consent. Trained study coordinators at each participating institution collected the data using a standardized format. Standardized definitions of all variables were determined by steering committee board of KAMIR-NIH.

### Percutaneous coronary intervention procedure and medical treatment

PCI for AMI was performed according to the standard practical guidelines, as previously described [14]. The application of predilatation, postdilatation and the selection of a specific type of implanted stent were at the discretion of the operator. Antiplatelet therapy and administration of periprocedural anticoagulation were carried out in accordance with the standard regimens. Aspirin (loading dose, 200mg) plus clopidogrel (loading dose, 300 or 600 mg) or ticagrelor (loading dose 180mg) or prasugrel (loading dose 60mg) were prescribed for all patient before or during PCI. After the procedure, aspirin (100-200mg/day) was maintained indefinitely. Patients with drug-eluting stents were prescribed clopidogrel (75mg/day), ticagrelor (90mg twice/day), prasugrel (10mg/day) for at least 12 months. Other cardiac medications were administered at the discretion of treating physicians.

### Definitions and study end-points

The diagnosis of AMI was based on detection of a raise and/or fall of cardiac biomarker (creatinine kinase-MB and troponin I or T) with at least one value above the 99th percentile upper reference limit and with at least one of the following: symptoms of ischemia, new or presumed new significant ST segment T wave changes or new left bundle branch block, development of pathological Q waves in the ECG, and imaging evidence of new loss of viable myocardium or new regional wall motion abnormality [15].

BMI was calculated at baseline by dividing the patient’s measured weight (in kg) by the square of the height (in m). The patients were divided into 3 groups stratified by BMI into quartiles: < 22 kg/m^2^ (Group 1), 22 to < 26 kg/m^2^ (Group 2), and ≥ 26 kg/m^2^ (Group 3) with supplementary grouping (Group 4: BMI ≥ 26 < 30 kg/m2) & (Group 5: BMI ≥30 kg/m2) for additional analysis. The glomerular filtration rate was calculated according to the abbreviated Modification of Diet and Renal Disease Study formula [16]. Chronic kidney disease (CKD) was defined as a glomerular filtration on admission of < 60ml/min per 1.73m^2^ [16]. Diabetes mellitus was defined as a fasting glucose concentration of ≥ 7.0 mmol/L, a blood glucose concentration of ≥ 11.0 mmol/L on a 75g, 2hr oral glucose tolerance test, or the use of antidiabetic therapy. Hypertension was defined as a history of a systolic blood pressure of ≥ 140mmHg, a diastolic pressure of ≥ 90mmHg, or the use of antihypertensive therapy. Hyperlipidemia was defined as a fasting total cholesterol concentration of ≥ 220mg/dL, a fasting triglyceride concentration of ≥ 150mg/dL, or the use of antihyperlipidemic therapy.

The primary outcome was an all cause death at 1 year. All deaths were considered as cardiac unless an unequivocal noncardiac cause could be established. Meanwhile, cardiac death (CD) was defined as any death due to a proximate cardiac cause such as myocardial infarction (MI), low-output failure, arrhythmia, unwitnessed death and all procedure-related deaths, including those related to conconmittant treatment [17]. MI was defined as newly developed Q wave, raised myocardial muscle creatinine kinase (CK-MB), Tn-I or T above the normal ranges, typical ischemic symptom accompanied with ST elevation. Target vessel revascularization (TVR) was defined as percutaneous or surgical revascularization of the stented lesion including 5mm margin segments and more proximal or distal, newly developed lesion. Also, cerebrovascular events were defined as a stroke or cerebrovascular accident with loss of neurological function caused by an ischemic or hemorrhagic event with residual symptoms at least 24 hours after onset or leading to death.

The other clinical outcome was heart failure, stent thrombosis and Thrombolysis in Myocardial Infarction (TIMI) minor bleeding [18] at 1 year. Heart failure was defined as sudden worsening of the signs and symptoms of heart failure, which was measured as an ejection fraction of less than 40% during follow-up. Stent thrombosis was evaluated according to the Academic Research Consortium Definitions [19]. TIMI minor bleeding was defined as overt clinical bleeding associated with a fall in hemoglobin of 3 to less than or equal to 5 g/dL (0.5 g/L) or in hematocrit of 9% to less than or equal to 15% (absolute). All adverse events were confirmed through the source documents, including medical records and telephone interviews, and were also adjudicated by the Steering Committee of Chonnam National University Hospital.

### Statistical analyses

Continuous variables were expressed as mean ± standard deviation, median (interquartile range) if strong positive skewness and categorical variables were presented as frequencies and percentage. Comparisons among the groups were calculated with an analysis of variance (ANOVA) and Kruskal-Wallis test for continuous variables and Pearson’s chi-square test for categorical variables. Also, post-hoc test was done with Bonferroni test. The incidences of the primary endpoints in the BMI groups were estimated at 12 month and were displayed in tables and Kaplan-Meier curves. The log-rank test was performed to compare the incidences of the endpoints between the 3 groups.

Meanwhile, Cox proportional hazard model was used to estimate the hazard ratio (HR) with 95% confidence interval (CI) for the statistical influence of low BMI after PCI on the clinical events. All of the variables in Tables 1 & 2 were included and analyzed to perform univariate analysis. The proportional hazard assumption was checked for all screened covariates, and no relevant violations were found. On the basis of the variables that were significant (*P* < 0.05) according to univariate analysis, a multivariate Cox proportional hazard model was constructed. Also, multivariable logistic regression analyses were carried out to identify independent predictors for overall mortality and on the basis of the variables that were significant (*P* < 0.05) according to univariable logistic regression analysis, a multivariable logistic regression model was performed. And, the consistency of treatment effects in subgroups was assessed with the use of Cox regression models with tests for interaction. P values <0.05 were considered as statistically significant. All statistical analyses were performed using a Statistical Analysis Software (SAS, version 9.2, SAS Institute, Cary, NC, USA).

**Table 1:** Baseline demographic, clinical and laboratory characteristics in patients with MI undergoing primary PCI stratified by BMI.

Variable	Group 1 N = 2632	Group 2 N = 5368	Group 3 N = 2568	P value
**Demographics**				
Age (years)	71 (61,78)^c^	63 (54,72)^b^	57 (49,67)^a^	<0.001
Male sex	1708 (64.9)	4266 (79.5)	2047 (79.7)	<0.001
BMI (kg/m2)	20.5 (19.3,21.3)^a^	23.9 (23.0,24.9)^b^	27.7 (26.8,29.3)^c^	<0.001
**Disease classification**				0.203
NSTEMI	1286 (48.9)	2526 (47.1)	1251 (48.7)	
STEMI	1346 (51.1)	2842 (52.9)	1317 (51.3)	
**Killip**				<0.001
I	1954 (74.2)	4342 (80.9)	2147 (83.6)	
II	262 (10)	414 (7.7)	179 (7)	
III	247 (9.4)	323 (6)	134 (5.2)	
IV	169 (6.4)	289 (5.4)	108 (4.2)	
**Risk factors**				
Family history of CAD	116 (4.4)	366 (6.8)	216 (8.4)	<0.001
Diabetes	733 (27.8)	1474 (27.5)	712 (27.7)	0.926
Hypertension	1282 (48.7)	2569 (47.9)	1403 (54.6)	<0.001
Hyperlipidemia	195 (7.4)	609 (11.3)	396 (15.4)	<0.001
Current/recent smoker	1389 (52.8)	3260 (60.7)	1654 (64.4)	<0.001
CKD (%)	598 (22.7)	909 (16.9)	108 (4.2)	<0.001
**Cardiovascular disease history**				
Prior myocardial infarction	156 (5.9)	296 (5.5)	162 (6.3)	0.352
Prior CHF	150 (5.7)	202 (3.8)	75 (2.9)	<0.001
Prior PCI	211 (8.0)	421 (7.8)	220 (8.6)	0.538
Atrial fibrillation/flutter	143 (5.4)	227 (4.2)	112 (4.4)	0.045
Cerebrovascular disease	199 (7.6)	313 (5.8)	128 (5.0)	<0.001
**Laboratory finding**				
HbA1c (%)	6.0 (5.5,7.0)^a^	6.1 (5.5,7.2)^a^	6.2 (5.6,7.3)^c^	<0.001
proBNP	987.5 (106.1,4495.1)^c^	320.4 (39.9,2701.2)^b^	185.3 (22.9,1943.2)^a^	<0.001
Hb (g/dL)	13.2 (11.6,14.5)^a^	14.3 (13.0,15.4)^b^	14.9 (13.6,15.9)^c^	<0.001
hsCRP (mg/L)	0.39 (0.06,2.41)^c^	0.30 (0.06,1.68)^a^	0.30 (0.07,1.37)^a^	<0.001
Total cholesterol (mg/dL)	169.0 (141.0,199.0)^a^	179.0 (151.6,209.0)^b^	185.0 (156.0,217.0)^c^	<0.001
Triglyceride (mg/dL)	86.0 (59.0,130.5)^a^	107.8 (72.0,165.0)^b^	132.1 (87.0,201.0)^c^	<0.001
LDL cholesterol (mg/dL)	104.6 (80.0,130.9)^a^	114.0 (88.0,139.3)^b^	117.0 (91.5,143.0)^c^	<0.001
HDL cholesterol (mg/dL)	43.2 (36.0,52.0)^c^	41.0 (35.0,48.0)^b^	40.0 (34.0,47.0)^a^	<0.001
**In-hospital medications**				
Aspirin	2631 (100.0)	5359 (99.8)	2567 (100.0)	0.120
Clopidogrel	2158 (82.0)	4096 (76.3)	1905 (74.2)	<0.001
Ticagrelor or prasugrel	788 (29.9)	1962 (36.5)	1035 (40.3)	<0.001
Beta blocker	2104 (79.9)	4575 (85.2)	2278 (88.7)	<0.001
Calcium channel blocker	139 (5.3)	270 (5.0)	178 (6.9)	0.002
ACE inhibitor or ARB	2024 (76.9)	4317 (80.4)	2145 (83.5)	<0.001
Statin	2385 (90.6)	5028 (93.7)	2435 (94.8)	<0.001
Oral anticoagulant (warfarin)	75 (2.8)	130 (2.4)	59 (2.3)	0.389
Gp IIb/IIIa inhibitor	324 (12.3)	810 (15.1)	467 (18.2)	<0.001
**LVEF < 40%, n (%)**	529 (20.1)	754 (14.0)	303 (11.8)	<0.001
**LVEF**	51.0 (43.0,58.0)^a^	53.0 (46.0,59.5)^b^	54.0 (47.0,60.2)^c^	<0.001

Data are presented as median (interquartile range), and number (percentage) where appropriate. Group was stratified by BMI quartiles (Group1 < 22 kg/m2, Group 2 ≥22 < 26 kg/m2 and Group 3 ≥26 kg/m2). In ANOVA analysis, values labeled with the different superscripts in a row indicate significant differences between groups based on Bonferroni’s multiple comparison test. BMI = body mass index, STEMI = ST segment elevation myocardial infarction, NSTEMI = non-ST segment elevation myocardial infarction, CAD = coronary artery disease, CKD = chronic kidney disease, CHF = congestive heart failure, PCI = percutaneous coronary intervention, Hb = hemoglobin, hsCRP = high sensitivity C-reactive protein, LDL cholesterol = low density lipoprotein cholesterol, HDL cholesterol = high density lipoprotein Cholesterol, ACE inhibitor = angiotensin-converting enzyme inhibitor, ARB = angiotensin receptor blocker, Gp IIb/IIIa inhibitor = glycoprotein IIb/IIIa inhibitor, LVEF = left ventricular ejection fraction

**Table 2:** Baseline angiographic characteristics in patients with MI undergoing primary PCI stratified by BMI.

Variable	Group 1	Group 2	Group 3	P value
**Target vessel (%)**				
LAD	1906 (72.4)	3806 (70.9)	1717 (66.9)	<0.001
LCX	1177 (44.7)	2325 (43.3)	1129 (44.0)	0.485
RCA	1418 (53.9)	2853 (53.1)	1377 (53.6)	0.811
LMCA	126 (4.8)	258 (4.8)	113 (4.4)	0.707
**Number of diseased vessels (%)**				0.054
1	1252 (47.6)	2641 (49.2)	1312 (51.1)	
2	804 (30.5)	1666 (31.0)	783 (30.5)	
3	535 (20.3)	973 (18.1)	434 (16.9)	
4	41 (1.6)	88 (1.6)	39 (1.5)	
**Lesion classification (%)**				
A	31 (1.6)	70 (1.3)	33 (1.3)	0.890
B1	321 (12.2)	644 (12.0)	284 (11.1)	0.378
B2	1001 (38.0)	1974 (36.8)	985 (38.4)	0.312
C	1279 (48.6)	2679 (49.9)	1266 (49.3)	0.538
**Pre-PCI TIMI 0 or 1, n (%)**	1442 (54.8)	3061 (57.0)	1517 (59.1)	0.008
**Post-PCI TIMI 0 or 1, n (%)**	10 (0.4)	14 (0.3)	7 (0.3)	0.635
**Post-PCI TIMI 3, n (%)**	2549 (96.8)	5217 (97.2)	2509 (97.7)	0.165
**Total number of stents (%)**	1.19 ± 0.43	1.18 ± 0.42	1.17 ± 0.41	0.303
**Total stent length**	29.87 ± 14.22	29.47 ± 14.27	29.14 ± 13.93	0.176
**Mean stent diameter**	2.98 ± 0.52^a^	3.05 ± 0.53^b^	3.11 ± 0.59^c^	<0.001

Data are presented as mean ± SD and number (percentage) where appropriate. Group was stratified by BMI quartiles (Group 1 < 22 kg/m2, Group 2 ≥22 < 26 kg/m2 and Group 3 ≥26 g/m2). Lesion based on American College of Cardiology/American Heart Association lesion classification. LAD = left anterior descending artery, LCX = left circumflex artery, LMCA = left main coronary artery, TIMI = Thrombolysis In Myocardial Infarction

## Results

### Baseline characteristics of study population

Baseline demographic, clinical and laboratory characteristics are presented according to the weight status (Table 1). Patients were classified into 3 groups according to the quartile: Group 1 (n = 2632, 24.9% of total subjects, BMI < 22 kg/m^2^), Group 2 (n = 5368, 50.8% of total subjects), Group 3 (n = 2568, 24.3% of total subjects). The population distributions for age, sex, Killip classification, family history of CAD, hypertension, hyperlipidemia, current/recent smoker, CKD, prior congestive heart failure (CHF), atrial fibrillation/flutter, cerebrovascular disease, HbA1c, proBNP, hemoglobin (Hb), hsCRP, lipid profile, the use of antiplatelet agents, beta blocker, calcium channel blocker, statin, glycoprotein (Gp) IIb/IIIa inhibitor and left ventricular ejection fraction (LVEF) significantly differed among the three groups. Group 1 was the oldest and Group 3 was the youngest. The proportion of male was the lowest in Group 1 and Group 1 showed higher incidences of aggravated heart failure indicating relatively higher proportion of killip classification of III and IV as well as pro-brain natriuretic peptide (proBNP) and LVEF < 40%. However, regarding risk factors, several factors such as CAD history, hypertension, hyperlipidemia and smoking history were significantly higher in Group 3 than those in Group 1 & 2, whereas CKD, prior CHF, atrial fibrillation/flutter and cerebrovascular disease in Group 2 were significantly higher than those in the other groups. Also, Hb in Group 1 was the lowest and hsCRP in the Group 1 was the highest among the groups. Meanwhile, although Group 3 showed significantly worse lipid profiles than other groups, the proportions of medications such as strong antiplatelet agents (ticagrelor or prasugrel), beta blocker, angiotensin-converting enzyme inhibitor (ACE inhibitor) or angiotensin II receptor blocker (ARB), statin and Gp IIb/IIIa inhibitor were relatively higher in Group 3 than in the other groups. There were no significant statistical differences between the three groups in other characteristics (Table 1).

The angiographic characteristics of Group 1 showed relatively higher proportion of LAD among target vessels and higher rates in the number of disease vessels. In addition, the mean stent diameter in Group 1 was the smallest among the groups, while there were no significant differences in the total number of stents and stent lengths among the groups (Table 2).

### Clinical outcomes of the study population

**Fig 2:**
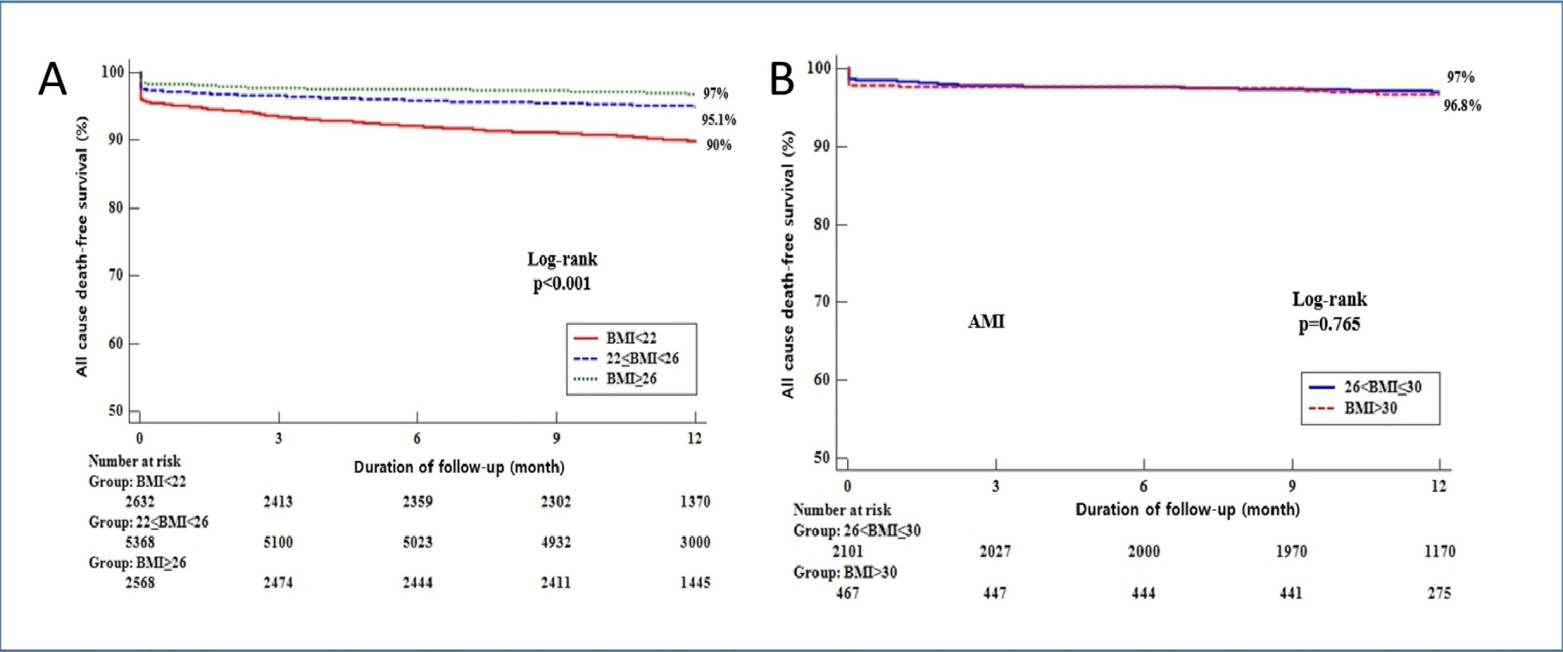
Kaplan-Meier Curve for the 12-month probability of all cause death-free survival in patients with MI undergoing primary PCI stratified by BMI. 12-month probability of all cause death-free survival stratified by BMI quartiles (A) (Group 1 < 22 kg/m2, Group 2 ≥ 22 < 26 kg/m2 and Group 3 ≥ 26 kg/m2) and (B) (Group 4 ≥ 26 < 30 kg/m2 and Group 5 ≥ 30 kg/m2).

The median follow-up duration was 12 month. Among the AMI patients undergoing PCI, classified by BMI, the cumulative rate of primary outcome for all cause death was significantly higher in the low BMI group (BMI < 22 kg/m^2^) than in the high BMI group (BMI ≥ 26 kg/m^2^) at 12 month (262 [10.0%] vs. 77 [3.0%], P<0.001, Table 3). Multivariate Cox regression analysis revealed that low BMI in AMI patients undergoing PCI is a predictor for these events (primary outcome, aHR 1.537 [1.177–2.007], P = 0.002 at 12 month, Table 3). In particular, these primary events would be driven by cardiac death (cardiac death, aHR 1.548 [1.128–2.124], P = 0.007 at 12 month, Table 3). Also, TIMI minor bleeding also occurred more frequently in the low BMI group (BMI < 22 kg/m^2^) than in the high BMI group (BMI ≥ 26 kg/m^2^) at 12 month (122 [4.6%] vs. 56 [2.2%], P<0.001; aHR 1.881 [1.337–2.646], P<0.001 at 12 month, S2 Table). Meanwhile, after further adjustment, stent thrombosis was associated with low BMI (BMI < 22 kg/m^2^), comparing to the high BMI group (BMI ≥ 26 kg/m^2^) at 12 month, that reached marginal significance (stent thrombosis, aHR 2.891 [0.949–8.812], P = 0.062 at 12 month, S2 Table). In addition, we analyzed the association between patients with BMI ≥26 < 30 kg/m^2^ and BMI ≥30 kg/m^2^ in this study. There was no significant differences in the primary outcome for all cause death (S1 Table). The event-free Kaplan-Meier curve for the primary outcome is shown in Fig 2B. Also, we analyzed the events in AMI patients not performing PCI, followed by no significant differences in the primary outcome as well as other individual components of outcomes (S4 Table).

**Table 3:** Clinical outcomes in AMI patients stratified by BMI at 1-year.

	Group		P-value	Log-rank P-value	HR	95% CI		P-value	Adjusted HR	95% CI		P-value
**1-year**												
**Primary** **end-point**			<0.001	<0.001								
**(All cause death)**	Group 1	262 (10.0)			3.421	2.653	4.411	<0.001	1.537	1.177	2.007	0.002
	Group 2	261 (4.9)		<0.001	1.634	1.267	2.107	<0.001	1.270	0.981	1.646	0.070
	Group 3	77 (3.0)		<0.001	1.000				1.000			
**Cardiac death**			<0.001	<0.001								
	Group 1	184 (7.0)			3.344	2.474	4.519	<0.001	1.548	1.128	2.124	0.007
	Group 2	55 (2.1)		<0.001	1.637	1.212	2.212	0.001	1.261	0.928	1.714	0.138
	Group 3	49 (1.9)		<0.001	1.000				1.000			
**Myocardial infarction**			0.196	0.433								
	Group 1	49 (1.9)			1.564	1.002	2.441	0.049	1.114	0.691	1.797	0.658
	Group 2	82 (1.5)		0.307	1.241	0.825	1.867	0.300	1.132	0.746	1.718	0.559
	Group 3	32 (1.2)		0.046	1.000				1.000			
**Target vessel revascularization**			0.259	0.508								
	Group 1	12 (0.5)			0.587	0.289	1.193	0.141	0.611	0.283	1.319	0.210
	Group 2	34 (0.6)		0.569	0.783	0.454	1.348	0.377	0.819	0.467	1.434	0.484
	Group 3	21 (0.8)		0.134	1.000				1.000			
**Cerebrovascular events**			0.032	0.005								
	Group 1	34 (1.3)			2.320	1.263	4.258	0.007	1.460	0.764	2.789	0.252
	Group 2	52 (1.0)		0.272	1.682	0.947	2.988	0.076	1.439	0.802	2.581	0.223
	Group 3	15 (0.6)		0.005	1.000				1.000			

Data are presented as n (%), CI, confidence interval; HR, hazard ratio. Group was stratified by BMI quartiles (Group 1 < 22 kg/m2, Group 2 ≥22 < 26 kg/m2 and Group 3 ≥26 kg/m2). All of the variables in Tables 1 & 2 were included and analyzed to perform univariate analysis. On the basis of the variables that were significant (*P* < 0.05) according to univariate analysis, a multivariate Cox proportional hazard model was constructed

### Predictors of the overall mortality

Univariable and multivariable logistic regression analyses were performed to identify independent predictors of mortality in patients with AMI undergoing PCI. In the multivariable logistic regression model, age, Killip classification, CKD, cerebrovascular disease, use of ß-blocker, ACE inhibitor/ARB, statin, Hb, HDL cholesterol, preprocedural suboptimal flow (TIMI 0 or 1 flow) and LVEF were independent predictors of overall mortality (Table 4).

**Table 4:** Multivariate analysis for overall mortality.

NSTEMI	Adjusted OR	95% CI		p-value
		Lower	Upper	
**Age**	1.045	1.034	1.057	<0.001
**Killip classification**	1.334	1.213	1.468	<0.001
**CKD**	1.335	1.046	1.703	0.020
**Cerebrovascular disease**	1.421	1.031	1.959	0.032
**Use of b-blocker**	0.405	0.322	0.511	<0.001
**Use of ACE inhibitor/ARB**	0.446	0.355	0.559	<0.001
**Use of statin**	0.170	0.133	0.217	<0.001
**Hb**	0.912	0.862	0.966	0.002
**HDL cholesterol**	0.990	0.981	1.000	0.044
**Pre-PCI TIMI 0 or 1**	0.884	0.811	0.963	0.005
**LVEF**	0.986	0.977	0.995	0.003

OR = odds ratio, CI = confidence interval, CKD = chronic kidney disease, ACE inhibitor = angiotensin-converting enzyme inhibitor, ARB = angiotensin receptor blocker, Hb = hemoglobin, HDL cholesterol = high density lipoprotein cholesterol, TIMI = Thrombolysis In Myocardial Infarction, LVEF = left ventricular ejection fraction. multivariable logistic regression analyses were carried out to identify independent predictors for overall mortality and on the basis of the variables that were significant (*P* < 0.05) according to univariable logistic regression analysis

### Subgroup analyses

**Fig 3:**
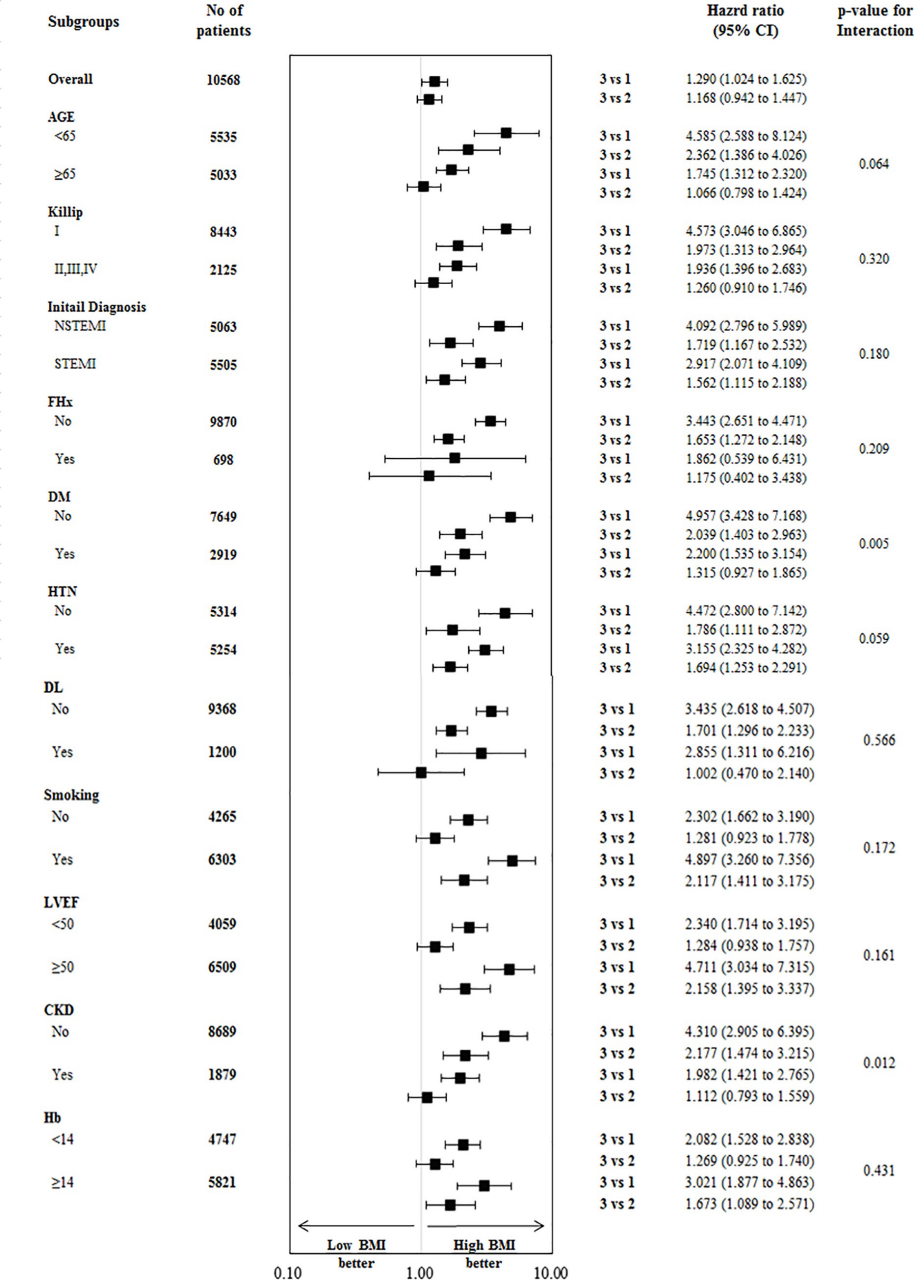
Subgroup analysis of the primary outcome. Hazard ratios and 95% confidence intervals are shown for the primary end point of all cause death in subgroups of patients assigned to BMI group. The P value for interaction represents the likelihood of interaction between the variable and the relative BMI effect.

The effect of BMI on the primary outcome was consistent across the subgroups except with respect to diabetes status and CKD, in which a trend toward a treatment-by-subgroup interaction was found (Fig 3) Among patients with diabetes, the rate of the primary outcome was significantly higher among those assigned to the low BMI group than among those assigned to the high BMI group (HR 2.200 [1.535–3.154], P<0.001, Fig 3). Among patients without diabetes, there was consistently significant difference in the rate of the primary outcome between the low BMI and the high BMI groups (HR 4.957 [3.428–7.168], P<0.001, Fig 3) (P = 0.005 for interaction). Meanwhile, among patients with CKD, the rate of the primary outcome was significantly higher among those assigned to the low BMI group than among those assigned to the high BMI group (HR 1.982 [1.421–2.765], P<0.001, Fig 3). Among patients without CKD, there was consistently significant difference in the rate of the primary outcome between the low BMI and the high BMI groups (HR 4.310 [2.905–6.395], P<0.001, Fig 3) (P = 0.012 for interaction). The interaction between the BMI groups, diabetes and renal status was also marginally observed with respect to the end points of age (P = 0.064 for interaction) and hypertension (P = 0.059 for interaction) (Fig 3).

## Discussion

The present study unearthed that the group with low BMI had poor clinical profiles including older age, a higher proportion of female, CKD, prior CHF, cerebrovascular disease and atrial fibrillation/flutter, lower Hb, higher inflammation marker as well as aggravated heart failure. On the other hand, although the group with high BMI had patients with younger age, a higher proportion of male and greater use of medications, it had relatively worse lipid profiles and risk factors including family history of CAD, hypertension, hyperlipidemia and current/recent smoker. Nevertheless, our clinical results demonstrated that the high BMI group is associated with better clinical outcomes in long-term follow-up in patients with AMI undergoing PCI.

The present study provides significant findings that AMI patients with low BMI had a significant correlation with the all cause death. Addressing this matter, our findings have important implications because we provide strong evidence with solid data and large sample size. First, the adverse associations of weighted-patients with adverse primary outcome were more prominent in patients with the lowest BMI quartile, compared to those with the highest BMI quartile. This result was consistent by the adverse effects of cardiac death, even after adjustments for several confounding factors (Table 3). More to the point, this study showed that the incidence of clinical outcomes according to the BMI quartiles was more prominent in the lowest BMI quartile than in the highest BMI quartile among all subgroups (Table 3 and Fig 3). Not only patients with STEMI but those with NSTEMI had consistent effects on primary endpoint and even patients with diabetes mellitus and CKD as well as old age and aggravated heart failure also showed comparable trends. Second, consistent with a previous finding [12], there was a lower percentage of acute anterior AMI presentation in patients with the higher BMI group (Table 2). Less incidence of anterior infarction in higher BMI group might affect favorable outcome in this study. Third, another remarkable points in the present study were proportion of CKD and level of Hb. A difference was already taken notice at the baseline characteristics (Table 1). One of the causes of anemia to take account is the prevalence of CKD across the BMI quartiles (Table 1), consistent to a previous study [12]. The higher incidences of anemia and CKD in the lowest BMI group could be strongly associated with poor clinical outcomes, as noted before [20, 21]. Fourth, patients with higher BMI quartiles were more frequently taking guideline-recommended therapies on admission including aspirin, new P2Y12 inhibitors (ticagrelor or prasugrel), b-blockers, ACE inhibitor or ARB, statin and Gp IIb/IIIa inhibitor. This wide-range use of optimal management in AMI patients undergoing PCI must be related with favorable results in patients with the higher BMI quartile. Fifth, one of crucial clinical factors influencing the results in the present study is age. Younger patients were more frequently observed in the higher BMI group. Previous studies demonstrated that coronary intervention in aged population is still challenging due to complex clinical situations such as comorbidities, side effects associated with multiple drug administration and greatly reduced cardiac function with serious coronary disease [22–24]. Our multivariate analysis (Table 4) showed the results consistent with the findings of previous studies. Advanced age, higher killip classification, lower left ventricular ejection fraction, a higher prevalence of CKD and a lower level of Hb as well as lower use of b-blocker, ACE inhibitor/ARB and statin were independent predictors of overall mortality. Additionally, we analyzed the association between patients with BMI ≥26 < 30 kg/m^2^ and BMI ≥30 kg/m^2^ in this study. Up to now, no study has been conducted in Asian countries to assess the patients with extremely higher BMI quartile (BMI > 30 or 35 kg/m^2^). Intriguingly, the individual components of other clinical outcome as well as primary end-point did not reach the significance between patients with BMI ≥26 < 30 kg/m^2^ and BMI ≥ 30 kg/m^2^ in this study (S1 Table and Fig 2B). Although our data showed no significant differences in outcomes (S1 Table), it should be taken into consideration for relatively small sample size. Nevertheless, the result in Asian population would warrant further trial for extremely obese population. Also, clinical outcomes in patients with MI not undergoing primary PCI or ballooning stratified by BMI showed interesting results. There were no significant differences not only in the event of all cause death but in the events of other clinical outcomes such as CD, MI, TVR, cerebrovascular events, heart failure and TIMI minor bleeding (S4 Table). Actually, this result would be driven from relatively small sample size. Accordingly, the analysis could be underpowered for detecting clinically relevant differences of clinical endpoints in this analysis. However, the discrepancy of clinical outcome subject to PCI or ballooning stratified by BMI would be worthwhile for further investigation.

Several potential mechanisms accounting for protective effects of obesity on clinical outcomes after AMI have been proposed; greater metabolic reserves, less cachexia, younger ages, more aggressive medical therapy, more aggressive diagnostic and revascularization procedures, increased muscle mass and strength, diminished hormonal response including the renin-angiotensin-aldosterone system, and unmeasured confounders, including selection bias [25]. Another possible explanation is that lower BMIs do not distinguish well between lean body mass and adipose tissue. It would be better predictive of amount of adipose tissue at higher BMIs [26]. Central adiposity or body fat content might be more clinically important than BMI, itself. The exact estimation of excess adipose tissue in accordance with weight status would be helpful to assess this issue.

There are several limitations in our study. First, although potentially clinical confounding covariates were adequately adjusted, the comparative results might be vulnerable to unmeasured confounders. Second, it was a nonrandomized observational registry and results are prone to be influenced by inherent limitations. Thus, large, prospective trials will be needed for further evaluation. Third, no consensus exists on what the optimal cutoff point of BMI is to determine obesity in Asians. Asians generally have a higher percentage of body fat than Europeans. Asian populations have also been shown to have an elevated risk of DM, hypertension, and hyperlipidemia at a relatively low level of BMI. Therefore, the BMI cutoff points for obesity should be lower for Asian populations than they are for European populations [27]. Fourth, we did not have the data related to the change in BMI or weight during the follow-up period. Therefore, we could not determine the impact of weight reduction on outcomes after PCI. Finally, the fact that the trial included only Korean populations could affect the generalizability of the findings.

## Conclusion

The current analysis of the KAMIR-NIH registry showed the clinical implications of BMI in patients with AMI undergoing PCI. Patients with higher levels of baseline BMI had better risk-profiles of baseline clinical, laboratory, and angiographic characteristics and also had favorable clinical outcomes. Further studies are required to address the optimal cut-off value in Asian population and long-term effects for patients with AMI and various BMI. Also, further clinical trials to evaluate beneficial effects of central obesity would be helpful to give an explanation of obesity paradox.

## Supporting information

S1 Fig Kaplan-Meier curve for the 12-month probability of all cause death-free survival in patients with MI not undergoing primary PCI stratified by BMI. (DOCX)

S1 Table Clinical outcomes between Group 4 (BMI ≥26 < 30 kg/m2) and Group 5 (BMI ≥30 kg/m2) in patients with MI undergoing primary PCI at 1-year. (DOCX)

S2 Table Other clinical outcomes in AMI patients stratified by BMI at 1-year. (DOCX)

S3 Table Baseline demographic, clinical and laboratory characteristics in patients with MI not undergoing primary PCI or ballooning stratified by BMI. (DOCX)

S4 Table Clinical outcomes in patients with MI not undergoing primary PCI or ballooning stratified by BMI. (DOCX)

S1 Dataset Raw clinical data with a fully anonymized data set. (XLSX)

## Abbreviations

CAD: coronary artery disease
DM: diabetes mellitus
BMI: body mass index
AMI: acute myocardial infarction
PCI: percutaneous coronary intervention
DES: drug-eluting stents
HR: hazard ratio
CI: confidence interval
CKD: chronic kidney disease
proBNP: pro B-type natriuretic peptide
Hb: hemoglobin
GpIIb/IIIa inhibitor: glycoprotein GpIIb/IIIa inhibitor
LVEF: left ventricular ejection fraction
ACE inhibitor: angiotensin-converting enzyme inhibitor
ARB: angiotensin II receptor blocker
