# Supplementary material for: Association between body mass index and 1-year outcome after acute myocardial infarction
Source: PLoS One. 2019 Jun 14;14(6):e0217525. doi: 10.1371/journal.pone.0217525 (PMC6570024; doi:10.1371/journal.pone.0217525)
Supplement: S1 Fig — (DOCX) [file pone.0217525.s001.docx]

**S1 Fig. Kaplan-Meier Curve for the 12-month probability of all cause death-free survival in patients with MI not undergoing primary PCI stratified by BMI.**


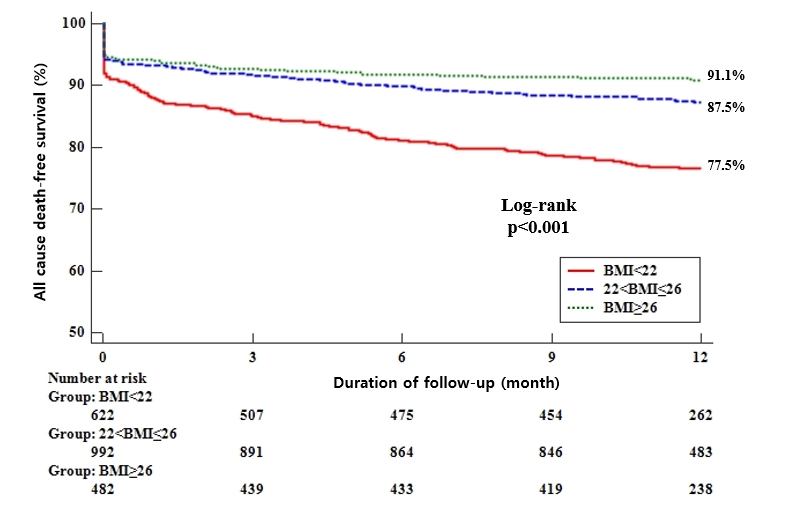


**Adjustment for multiple comparisons for the log-rank test with Bonferroni correction**

**Group was stratified by BMI quartiles (Group Ia < 22 kg/m2, Group IIa ≥22 < 26 kg/m2 and Group IIIa ≥26 kg/m2).**

**Group 1 vs 2, p <0.001; Group 1 vs 3, p <0.001; Group 2 vs 3, p = 0.674**
