# Supplementary material for: Association between body mass index and 1-year outcome after acute myocardial infarction
Source: PLoS One. 2019 Jun 14;14(6):e0217525. doi: 10.1371/journal.pone.0217525 (PMC6570024; doi:10.1371/journal.pone.0217525)
Supplement: S1 Table — (DOCX) [file pone.0217525.s002.docx]

**S1 Table. Clinical outcomes between Group 4 (BMI ≥26 < 30 kg/m2) and Group 5 (BMI ≥30 kg/m2) in patients with MI undergoing primary PCI stratified at 1-year.**

|  |  | |  |  |  | |  | |  | |  | |  |  | |  | |  | |  |
| --- | --- | --- | --- | --- | --- | --- | --- | --- | --- | --- | --- | --- | --- | --- | --- | --- | --- | --- | --- | --- |
|  | **Group** |  | **P**  **value** | **Log-rank**  **p-value** | | **HR** | | **95.0% CI** | | | | **p**  **value** | **Adjusted**  **HR** | | **95.0% CI** | | | | **p**  **value** | |
|  |  |  |  |  |  |  |  |  | |  | |  |  |  |  | |  | |  |  |
| **1-year** |  |  |  |  |  | |  | |  | |  | |  |  | |  | |  | |  |
| **Primary**  **end-point** |  |  | 0.765 | 0.765 |  | |  | |  | |  | |  |  | |  | |  | |  |
| **(All cause death)** | Group 4 | 62 (3.0) |  |  |  | |  | |  | |  | |  |  | |  | |  | |  |
|  | Group 5 | 15 (3.2) |  |  | 1.090 | | 0.620 | | 1.915 | | 0.766 | | 1.243 | 0.701 | | 2.204 | | 0.456 | |  |
| **Cardiac death** |  |  | 0.480 | 0.479 |  | |  | |  | |  | |  |  | |  | |  | |  |
|  | Group 4 | 43 (2.0) |  |  |  | |  | |  | |  | |  |  | |  | |  | |  |
|  | Group 5 | 12 (2.6) |  |  | 1.259 | | 0.664 | | 2.387 | | 0.481 | | 1.475 | 0.769 | | 2.829 | | 0.242 | |  |
| **Myocardial infarction** |  |  | 0.194 | 0.196 |  | |  | |  | |  | |  |  | |  | |  | |  |
|  | Group 4 | 29 (1.4) |  |  |  | |  | |  | |  | |  |  | |  | |  | |  |
|  | Group 5 | 3 (0.6) |  |  | 0.465 | | 0.142 | | 1.527 | | 0.207 | | 0.497 | 0.150 | | 1.647 | | 0.253 | |  |
| **Target vessel revascularization** |  |  | 0.918 | 0.921 |  | |  | |  | |  | |  |  | |  | |  | |  |
|  | Group 4 | 17 (0.8) |  |  |  | |  | |  | |  | |  |  | |  | |  | |  |
|  | Group 5 | 4 (0.9) |  |  | 1.056 | | 0.355 | | 3.140 | | 0.921 | | 1.011 | 0.331 | | 3.086 | | 0.984 | |  |
| **Cerebrovascular events** |  |  | 0.497 | 0.393 |  | |  | |  | |  | |  |  | |  | |  | |  |
|  | Group 4 | 11 (0.5) |  |  |  | |  | |  | |  | |  |  | |  | |  | |  |
|  | Group 5 | 4 (0.9) |  |  | 1.639 | | 0.522 | | 5.147 | | 0.397 | | 1.688 | 0.526 | | 5.420 | | 0.379 | |  |
| **Heart failure** |  |  | 0.913 | 0.913 |  | |  | |  | |  | |  |  | |  | |  | |  |
|  | Group 4 | 61 (2.9) |  |  |  | |  | |  | |  | |  |  | |  | |  | |  |
|  | Group 5 | 14 (3.0) |  |  | 1.033 | | 0.578 | | 1.846 | | 0.913 | | 1.158 | 0.644 | | 2.084 | | 0.624 | |  |
| **Stent thrombosis** |  |  | 0.592 | 0.293 |  | |  | |  | |  | |  |  | |  | |  | |  |
|  | Group 4 | 5 (0.2) |  |  |  | |  | |  | |  | |  |  | |  | |  | |  |
|  | Group 5 | 0 (0.0) |  |  | - | | - | | - | | - | | - | - | | - | | - | |  |
| **TIMI minor bleeding** |  |  | 0.265 | 0.265 |  | |  | |  | |  | |  |  | |  | |  | |  |
|  | Group 4 | 49 (2.3) |  |  |  | |  | |  | |  | |  |  | |  | |  | |  |
|  | Group 5 | 7 (1.5) |  |  | 0.643 | | 0.291 | | 1.419 | | 0.274 | | 0.613 | 0.275 | | 1.367 | | 0.232 | |  |

Data are presented as n (%), CI, confidence interval; HR, hazard ratio

Group 4 (BMI ≥26 < 30 kg/m2) : reference

Group was stratified by BMI quartiles (Group 4: BMI ≥26 < 30 kg/m2, Group 5: BMI ≥30 kg/m2).
