# Supplementary material for: Association between body mass index and 1-year outcome after acute myocardial infarction
Source: PLoS One. 2019 Jun 14;14(6):e0217525. doi: 10.1371/journal.pone.0217525 (PMC6570024; doi:10.1371/journal.pone.0217525)
Supplement: S2 Table — (DOCX) [file pone.0217525.s003.docx]

**S2 Table. Other clinical outcomes in AMI patients stratified by BMI at 1-year.**

|  |  | |  |  |  | |  | |  | |  | |  | |  | |  | |  | |  |
| --- | --- | --- | --- | --- | --- | --- | --- | --- | --- | --- | --- | --- | --- | --- | --- | --- | --- | --- | --- | --- | --- |
|  | **Group** |  | **p-value** | **Log-rank**  **p-value** | | **HR** | | **95.0% CI** | | | | **p-value** | | **aHR** | | **95.0% CI** | | | | **p-value** | |
|  |  |  |  |  |  |  |  |  | |  | |  |  |  |  |  | |  | |  |  |
| **1-year** |  |  |  |  |  | |  | |  | |  | |  | |  | |  | |  | |  |
| **Heart failure** |  |  | <0.001 | <0.001 |  | |  | |  | |  | |  | |  | |  | |  | |  |
|  | Group 1 | 150 (5.7) |  |  | 1.952 | | 1.480 | | 2.576 | | <0.001 | | 0.988 | | 0.729 | | 1.340 | | 0.940 | |  |
|  | Group 2 | 202 (3.8) |  | <0.001 | 1.289 | | 0.989 | | 1.680 | | 0.061 | | 1.000 | | 0.757 | | 1.322 | | 0.997 | |  |
|  | Group 3 | 75 (2.9) |  | <0.001 | 1.000 | |  | |  | |  | | 1.000 | |  | |  | |  | |  |
| **Stent thrombosis** |  |  | 0.166 | 0.463 |  | |  | |  | |  | |  | |  | |  | |  | |  |
|  | Group 1 | 13 (0.5) |  |  | 2.638 | | 0.940 | | 7.399 | | 0.065 | | 2.891 | | 0.949 | | 8.812 | | 0.062 | |  |
|  | Group 2 | 17 (0.3) |  | 0.275 | 1.645 | | 0.607 | | 4.459 | | 0.328 | | 1.799 | | 0.649 | | 4.985 | | 0.259 | |  |
|  | Group 3 | 5 (0.2) |  | 0.050 | 1.000 | |  | |  | |  | | 1.000 | |  | |  | |  | |  |
| **TIMI minor**  **bleeding** |  |  | <0.001 | <0.001 |  | |  | |  | |  | |  | |  | |  | |  | |  |
|  | Group 1 | 122 (4.6) |  |  | 2.129 | | 1.551 | | 2.921 | | <0.001 | | 1.881 | | 1.337 | | 2.646 | | <0.001 | |  |
|  | Group 2 | 155 (2.9) |  | <0.001 | 1.325 | | 0.976 | | 1.799 | | 0.071 | | 1.279 | | 0.937 | | 1.746 | | 0.121 | |  |
|  | Group 3 | 56 (2.2) |  | <0.001 | 1.000 | |  | |  | |  | | 1.000 | |  | |  | |  | |  |

Data are presented as n (%), CI, confidence interval; HR, hazard ratio, aHR, adjusted hazard ratio

Group was stratified by BMI quartiles (Group I < 22 kg/m2, Group II ≥22 < 26 kg/m2 and Group III ≥26 kg/m2).

TIMI = Thrombolysis In Myocardial Infarction

All of the variables in Table 1 & 2 were included and analyzed to perform univariate analysis. On the basis of the variables

that were significant (*P* < 0.05) according to univariate analysis, a multivariate Cox proportional hazard model was

constructed
