# Supplementary material for: Association between body mass index and 1-year outcome after acute myocardial infarction
Source: PLoS One. 2019 Jun 14;14(6):e0217525. doi: 10.1371/journal.pone.0217525 (PMC6570024; doi:10.1371/journal.pone.0217525)
Supplement: S3 Table — (DOCX) [file pone.0217525.s004.docx]

**S3 Table. Baseline demographic, clinical and laboratory characteristics in patients with MI not undergoing primary PCI or ballooning stratified by BMI.**

| **Variable** | **Group Ia**  **N=622** | **Group IIa**  **N=992** | **Group IIIa**  **N=482** | | **P value** | |
| --- | --- | --- | --- | --- | --- | --- |
| **Demographics** |  |  |  | |  | |
| Age (years) | 73 (65,80)^c^ | 65 (56,74)^b^ | 61 (51,72)^a^ | | <0.001 | |
| Male sex | 356 (57.2) | 702 (70.8) | 335 (69.5) | | <0.001 | |
| BMI (kg/m2) | 20.2 (18.7,21.1)^a^ | 23.9 (23.0,24.9)^b^ | 27.7 (26.8,29.4)^c^ | | <0.001 | |
| **Disease classification** |  |  |  | | 0.019 | |
| NSTEMI | 478 (76.8) | 729 (73.5) | 334 (69.3) | |  | |
| STEMI | 144 (23.2) | 263 (26.5) | 148 (30.7) | |  | |
| **Killip** |  |  |  | | <0.001 | |
| I | 405 (65.1) | 739 (74.5) | 381 (79) | |  | |
| II | 91 (14.6) | 100 (10.1) | 41 (8.5) | |  | |
| III | 92 (14.8) | 104 (10.5) | 39 (8.1) | |  | |
| IV | 34 (5.5) | 49 (4.9) | 21 (4.4) | |  | |
| **Risk factors** |  |  |  | |  | |
| Family history of CAD | 22 (3.5) | 60 (6.0) | 30 (6.2) | | 0.057 | |
| Diabetes | 210 (33.8) | 325 (32.8) | 169 (35.1) | | 0.677 | |
| Hypertension | 341 (54.8) | 530 (53.4) | 317 (65.8) | | <0.001 | |
| Hyperlipidemia | 43 (6.9) | 121 (12.2) | 79 (16.4) | | <0.001 | |
| Current/recent smoker | 280 (45.0) | 523 (52.7) | 274 (56.8) | | <0.001 | |
| CKD (%) | 211 (33.9) | 248 (25.0) | 116 (24.1) | | <0.001 | |
| **Cardiovascular disease history** |  |  |  | |  | |
| Prior myocardial infarction | 107 (17.2) | 185 (18.6) | 85 (17.6) | | 0.743 | |
| Prior CHF | 47 (7.6) | 55 (5.5) | 29 (6.0) | | 0.259 | |
| Prior PCI | 148 (23.8) | 267 (26.9) | 120 (24.9) | | 0.352 | |
| Atrial fibrillation/flutter | 65 (10.5) | 88 (8.9) | 41 (8.5) | | 0.460 | |
| Cerebrovascular disease | 55 (8.8) | 99 (10.0) | 56 (11.6) | | 0.313 | |
| **Laboratory finding** |  |  |  | |  | |
| HbA1c (%) | 6.1 (5.5,6.9) | 6.2 (5.6,7.2) | 6.2 (5.6,7.2) | | 0.227 | |
| proBNP | 3211.0 (338.3,10143.0)^b^ | 745.5 (80.0,5892.2)^a^ | 470.9 (41.6,4532.0)^a^ | | <0.001 | |
| Hb (g/dL) | 12.3 (10.7,13.9)^a^ | 13.7 (12.1,14.9)^b^ | 14.1 (12.5,15.4)^c^ | | <0.001 | |
| hsCRP (mg/L) | 0.93 (0.08,3.54)^b^ | 0.44 (0.06,2.74)^a^ | 0.31 (0.08,1.80)^a^ | | <0.001 | |
| Total cholesterol (mg/dL) | 156.5 (127.0,184.0)^a^ | 163.0 (130.0,197.0)^b^ | 167.5 (140.0,197.0)^b^ | | <0.001 | |
| Triglyceride (mg/dL) | 84.0 (58.0,123.0)^a^ | 101.5 (69.0,157.0)^b^ | 120.5 (80.0,183.2)^b^ | | <0.001 | |
| LDL cholesterol (mg/dL) | 90.1 (68.0,118.1)^a^ | 97.2 (71.0,125.3)^b^ | 99.2 (76.0,125.0)^b^ | <0.001 | |  |
| HDL cholesterol (mg/dL) | 44.0 (34.2,55.1)^b^ | 42.1 (34.0,51.0)^a^ | 42.0 (35.0,49.1)^a^ | | <0.001 | |
| **In-hospital medications** |  |  |  | |  | |
| Aspirin | 613 (98.6) | 970 (97.8) | 472 (97.9) | | 0.541 | |
| Clopidogrel | 555 (89.2) | 815 (82.2) | 391 (81.1) | | <0.001 | |
| Ticagrelor or prasugrel | 93 (15.0) | 226 (22.8) | 134 (27.8) | | <0.001 | |
| Beta blocker | 374 (60.1) | 678 (68.3) | 333 (69.1) | | <0.001 | |
| Calcium channel blocker | 107 (17.2) | 221 (22.3) | 119 (24.7) | | 0.006 | |
| ACE inhibitor or ARB | 361 (58.0) | 626 (63.1) | 336 (69.7) | | <0.001 | |
| Statin | 481 (77.3) | 796 (80.2) | 415 (86.1) | | 0.001 | |
| Oral anticoagulant (warfarin) | 34 (5.5) | 68 (6.9) | 32 (6.6) | | 0.523 | |
| Gp IIb/IIIa inhibitor | 29 (4.7) | 75 (7.6) | 43 (8.9) | | 0.015 | |
| **LVEF < 40%, n (%)** | 189 (30.4) | 220 (22.2) | 85 (17.6) | | <0.001 | |
| **LVEF** | 51.0 (40.0,60.0)^a^ | 54.0 (44.0,62.0)^b^ | 55.0 (46.9,62.0)8^b^ | | <0.001 | |

Data are presented as median (interquartile range), and number (percentage) where appropriate. Group was stratified by BMI quartiles (Group1a < 22 kg/m2, Group 2a ≥22 < 26 kg/m2 and Group 3a ≥26 kg/m2). In ANOVA analysis, values labeled with the different superscripts in a row indicate significant differences between groups based on Bonferroni’s multiple comparison test.

BMI = body mass index, STEMI = ST segment elevation myocardial infarction, NSTEMI = non-ST segment

elevation myocardial infarction, CAD = coronary artery disease, CKD = chronic kidney disease, CHF =

congestive heart failure, PCI = percutaneous coronary intervention, Hb = hemoglobin, hsCRP = high sensitivity

C-reactive protein, LDL cholesterol = low density lipoprotein cholesterol, HDL cholesterol = high density

Lipoprotein Cholesterol, ACE inhibitor = angiotensin-converting enzyme inhibitor, ARB = angiotensin receptor

blocker, Gp IIb/IIIa inhibitor = glycoprotein IIb/IIIa inhibitor, LVEF = left ventricular ejection fraction
