# Supplementary material for: Association between body mass index and 1-year outcome after acute myocardial infarction
Source: PLoS One. 2019 Jun 14;14(6):e0217525. doi: 10.1371/journal.pone.0217525 (PMC6570024; doi:10.1371/journal.pone.0217525)
Supplement: S4 Table — (DOCX) [file pone.0217525.s005.docx]

**S4 Table. Clinical outcomes in patients with MI not undergoing primary PCI or ballooning stratified by BMI.**

|  |  | |  |  |  | |  | |  | |  | |  |  | |  | |  | |  |
| --- | --- | --- | --- | --- | --- | --- | --- | --- | --- | --- | --- | --- | --- | --- | --- | --- | --- | --- | --- | --- |
|  | **Group** |  | **p-value** | **Log-rank**  **p-value** | | **HR** | | **95.0% CI** | | | | **p-value** | **Adjusted**  **HR** | | **95.0% CI** | | | | **p-value** | |
|  |  |  |  |  |  |  |  | **Lower** | | **Upper** | |  |  |  | **Lower** | | **Upper** | |  |  |
| **1-year** |  |  |  |  |  | |  | |  | |  | |  |  | |  | |  | |  |
| **Primary**  **end-point** |  |  | <0.001 | <0.001 |  | |  | |  | |  | |  |  | |  | |  | |  |
| **(All cause death)** | Group 1a | 140 (22.5) |  |  | 2.701 | | 1.919 | | 3.801 | | <0.001 | | 1.220 | 0.845 | | 1.762 | | 0.288 | |  |
|  | Group 2a | 124 (12.5) |  | <0.001 | 1.415 | | 1.000 | | 2.001 | | 0.050 | | 1.044 | 0.731 | | 1.490 | | 0.812 | |  |
|  | Group 3a | 43 (8.9) |  | <0.001 | 1.000 | |  | |  | |  | | 1.000 |  | |  | |  | |  |
| **Cardiac death** |  |  | <0.001 | <0.001 |  | |  | |  | |  | |  |  | |  | |  | |  |
|  | Group 1a | 98 (15.8) |  |  | 2.431 | | 1.638 | | 3.607 | | <0.001 | | 1.054 | 0.688 | | 1.612 | | 0.810 | |  |
|  | Group 2a | 98 (9.9) |  | <0.001 | 1.454 | | 0.980 | | 2.157 | | 0.063 | | 1.101 | 0.734 | | 1.652 | | 0.641 | |  |
|  | Group 3a | 33 (6.8) |  | <0.001 | 1.000 | |  | |  | |  | | 1.000 |  | |  | |  | |  |
| **Myocardial infarction** |  |  | 0.439 | 0.510 |  | |  | |  | |  | |  |  | |  | |  | |  |
|  | Group 1a | 13 (2.1) |  |  | 0.729 | | 0.347 | | 1.533 | | 0.405 | | 0.614 | 0.269 | | 1.401 | | 0.247 | |  |
|  | Group 2a | 21 (2.1) |  | 0.751 | 0.685 | | 0.353 | | 1.330 | | 0.264 | | 0.637 | 0.320 | | 1.268 | | 0.199 | |  |
|  | Group 3a | 15 (3.1) |  | 0.450 | 1.000 | |  | |  | |  | | 1.000 |  | |  | |  | |  |
| **TVR** |  |  | 0.624 | 0.621 |  | |  | |  | |  | |  |  | |  | |  | |  |
|  | Group 1a | 1 (0.2) |  |  | - | | - | | - | | - | | - | - | | - | | - | |  |
|  | Group 2a | 2 (0.2) |  | 0.782 | - | | - | | - | | - | | - | - | | - | | - | |  |
|  | Group 3a | 0 (0.0) |  | 0.490 | - | | - | | - | | - | | - | - | | - | | - | |  |
| **Cerebrovascular events** |  |  | 0.248 | 0.186 |  | |  | |  | |  | |  |  | |  | |  | |  |
|  | Group 1a | 11 (1.8) |  |  | 3.142 | | 0.876 | | 11.26 | | 0.079 | | 3.212 | 0.736 | | 14.01 | | 0.121 | |  |
|  | Group 2a | 14 (1.4) |  | 0.545 | 2.306 | | 0.663 | | 8.025 | | 0.189 | | 2.977 | 0.753 | | 11.77 | | 0.120 | |  |
|  | Group 3a | 3 (0.6) |  | 0.070 | 1.000 | |  | |  | |  | | 1.000 |  | |  | |  | |  |
| **Heart failure** |  |  | <0.001 | <0.001 |  | |  | |  | |  | |  |  | |  | |  | |  |
|  | Group 1a | 47 (7.6) |  |  | 1.256 | | 0.791 | | 1.995 | | 0.335 | | 0.720 | 0.436 | | 1.190 | | 0.200 | |  |
|  | Group 2a | 55 (5.5) |  | 0.112 | 0.921 | | 0.588 | | 1.445 | | 0.722 | | 0.812 | 0.509 | | 1.294 | | 0.381 | |  |
|  | Group 3a | 29 (6.0) |  | 0.248 | 1.000 | |  | |  | |  | | 1.000 |  | |  | |  | |  |
| **Stent thrombosis** |  |  | 0.540 | 0.562 |  | |  | |  | |  | |  |  | |  | |  | |  |
|  | Group 1a | 0 (0.0) |  |  | - | | - | | - | | - | | - | - | | - | | - | |  |
|  | Group 2a | 1 (0.1) |  | 0.620 | 0.489 | | 0.031 | | 7.824 | | 0.613 | | - | - | | - | | - | |  |
|  | Group 3a | 1 (0.2) |  | 0.283 | 1.000 | |  | |  | |  | | - | - | | - | | - | |  |
| **TIMI minor** |  |  | 0.232 | 0.232 |  | |  | |  | |  | |  |  | |  | |  | |  |
| **bleeding** | Group 1a | 25 (4.0) |  |  | 1.761 | | 0.867 | | 3.579 | | 0.118 | | 1.286 | 0.592 | | 2.793 | | 0.525 | |  |
|  | Group 2a | 29 (2.9) |  | 0.273 | 1.281 | | 0.640 | | 2.564 | | 0.484 | | 1.165 | 0.569 | | 2.384 | | 0.676 | |  |
|  | Group 3a | 11 (2.3) |  | 0.092 | 1.000 | |  | |  | |  | | 1.000 |  | |  | |  | |  |

Data are presented as n (%), CI, confidence interval; HR, hazard ratio; TVR, target vessel revascularization

Group was stratified by BMI quartiles (Group Ia < 22 kg/m2, Group IIa ≥22 < 26 kg/m2 and Group IIIa ≥26 kg/m2).

All of the variables in Table 1 & 2 were included and analyzed to perform univariate analysis. On the basis of the

variables that were significant (*P* < 0.05) according to univariate analysis, a multivariate Cox proportional hazard

model was constructed
